# Prolonged Time to Surgery in Patients With Residual Disease After Neoadjuvant Chemoradiotherapy for Esophageal Cancer

**DOI:** 10.1097/SLA.0000000000006488

**Published:** 2024-08-13

**Authors:** Hidde C.G. Overtoom, Ben M. Eyck, Berend J. van der Wilk, Bo J. Noordman, Pieter C. van der Sluis, Bas P.L. Wijnhoven, J. Jan B. van Lanschot, Sjoerd M. Lagarde

**Affiliations:** Department of Surgery, Erasmus MC Cancer Institute, Erasmus University Medical Center, Rotterdam, The Netherlands

**Keywords:** esophageal cancer, neoadjuvant chemoradiotherapy, prolonged time to surgery, residual disease

## Abstract

**Objective:**

To investigate whether prolonged time to surgery negatively affects survival, pathologic outcome, or postoperative complications in patients with histologically proven residual disease after neoadjuvant chemoradiotherapy (nCRT) for locally advanced esophageal cancer.

**Background:**

Historically, the standard time to surgery (TTS) has been 6 to 8 weeks after completion of nCRT. The effect of prolonged TTS is gaining interest, with contradicting results on survival and surgical morbidity. It can be hypothesized that, in patients with residual disease 6 weeks after completion of nCRT, prolonged TTS might be associated with worse survival and higher morbidity.

**Methods:**

Patients with locally advanced esophageal cancer who had biopsy-proven residual disease 6 weeks after nCRT and underwent surgery, were categorized according to interval to surgery (TTS>12w vs TTS≤12w). The primary outcome of this study was overall survival. Secondary outcomes were disease-free survival, surgical outcomes, pathologic outcomes, and postoperative complications. Multivariable Cox regression was used for comparing survival and logistic regression for other outcomes, adjusted for the confounders age, cT, cN, Charlson comorbidity index, weight loss during nCRT, and WHO performance score after completion of nCRT.

**Results:**

Forty patients were included for TTS>12w and 127 for TTS≤12w. TTS>12w was associated with better overall survival [adjusted hazard ratio (aHR) 0.46, 95% CI: 0.24–0.90], and disease-free survival (aHR 0.48, 95% CI: 0.24–0.94), but also with more postoperative respiratory complications (aOR 3.66, 95% CI: 1.52–9.59). Other outcomes were comparable between both groups.

**Conclusions:**

Prolonged TTS in patients with histologically proven residual disease after completion of nCRT for esophageal cancer did not have a negative effect on overall and disease-free survival, but patients did have a higher risk for postoperative respiratory complications.

In many countries, the standard treatment of locally advanced esophageal cancer (LAEC) is neoadjuvant chemoradiotherapy (nCRT) followed by esophagectomy.^1,2^ Historically, the standard time to surgery (TTS) has been 6 to 8 weeks after completion of nCRT. Since the first publication on this subject by Kim et al,^3^ the effect of prolonged TTS for LAEC is gaining interest, with contradicting results on survival and surgical morbidity.^4–7^

To improve health-related quality of life, organ-sparing strategies are gaining increasing attention for patients with a clinically complete response to nCRT.^8–10^ In the SANO (Surgery as Needed for Oesophageal cancer) trial, clinical response evaluations are performed to detect residual disease after nCRT.^10–12^ Early clinical response evaluation 6 weeks after nCRT is performed to identify patients with a poor response to nCRT to prevent unnecessary delay of esophagectomy and is believed to maximize the probability of curation. Surgery after detection of residual disease, however, is not always possible due to several reasons, among others poor physical condition and logistic delay. It can be hypothesized that in those patients who have residual disease, a prolonged time to surgery might well have negative effects on survival. Therefore, the aim of the present study was to investigate whether prolonged TTS has a negative effect on survival, pathologic outcome, or postoperative complications in patients with histologically proven residual disease 6 weeks after completion of nCRT.

## METHODS

### Study Design and Patients

This multicenter retrospective cohort study was performed using the preSANO and SANO trial data, obtained from centers participating in the SANO trial.^11–13^ The preSANO trial is a prospective diagnostic cohort study that included patients from July 2013 through December 2016.^13^ The SANO trial is a phase 3 stepped-wedge cluster randomized controlled trial that included patients from November 2017 through May 2021.^11,12^ For the present study, patients who completed nCRT before June 2020 were included.

In both the preSANO and SANO trials, patients with LAEC (cT1N1-3M0 or cT2-4aN0-3M0 according to UICC TNM staging manual, seventh edition) were included. All patients were treated with nCRT according to the CROSS regimen and underwent esophagogastroduodenoscopy with biopsies 4 to 6 weeks after completion of nCRT.^12,14,15^ For the present study, patients were eligible if they completed at least 80% of neoadjuvant chemotherapy, and if residual disease was histopathologically confirmed in the endoscopic biopsies taken 4 to 6 weeks after nCRT. Patients were excluded if no preoperative positron emission tomography/computed tomography scan (PET/CT) was performed, or if preoperative PET/CT showed distant metastases. All patients provided written informed consent. The Medical Ethical Committee of the Erasmus Medical Center has approved the study protocol (MEC-2020-0958).

### Neoadjuvant Chemoradiotherapy

The CROSS regimen consists of 5 weekly cycles of intravenous paclitaxel (50 mg/m^2^) and carboplatin (area under the curve (AUC) 2 mg/mL/min). Concurrently, a total dose of 41.4 Gy external beam radiotherapy was given in 23 fractions of 1.8 Gy for 5 days per week, starting on the first day of each treatment cycle.^14^

### Clinical Response Evaluation

During the esophagogastroduodenoscopy 4 to 6 weeks after completion of nCRT, the esophagus and esophagogastric junction, including the primary tumor location, were assessed for suspicious lesions. All patients should have had at least 4 biopsies of the primary tumor location, as well as biopsies of any suspicious lesion. Both regular and bite-on-bite biopsy techniques (keyhole biopsies with conventional biopsy forceps) were considered eligible. In case of a post-chemoradiotherapy ulcer or erosion, biopsies were taken at the borders of the ulcer.

Patients underwent a PET/CT if histopathologic evidence of residual tumor in the biopsies was obtained to exclude distant interval metastases before esophagectomy was planned. PET/CT was performed according to the European Association of Nuclear Medicine guidelines and reviewed by an expert nuclear medicine physician.^16^

### Surgery

Surgical approach depended on patient and tumor characteristics and local expertise. A transthoracic or transhiatal approach, aiming at resection of the primary tumor with at least 15 regional lymph nodes, was performed. Both approaches included a dissection of lymph nodes around the primary tumor and around the celiac trunk. In the transthoracic approach, an additional mediastinal lymph node dissection was performed. Surgical techniques included open, totally minimally invasive, or hybrid minimally invasive surgery. For reconstruction, a gastric conduit was preferred.

### Pathology

All biopsies were analyzed based on regular hematoxylin and eosin slides at 2 or 3 levels. If analysis at these levels did not reveal obvious high-grade dysplasia or vital tumor, 1 to 3 additional deeper sections were taken, depending on the amount of tissue in the paraffin block. In case the presence of tumor cells was still uncertain at these additional levels, dPAS and (pan)keratin staining were performed. Histopathologic outcome was considered negative if no high-grade dysplasia or tumor cells were detected during these additional sections and stainings.

Pathologic examination of the resection specimen was performed by expert gastrointestinal pathologists following a standard protocol. If the presence of tumor cells remained uncertain, a second gastrointestinal pathologist was consulted. In case no consensus was reached, a third pathologist was included for the final conclusion. Pathologic staging was performed according to the seventh edition of the UICC TNM staging manual.^17^ Microscopically radical (R0) resection was defined as a tumor-free proximal, distal, and circumferential resection margin (margin >1 mm not required). Tumor regression grade (TRG) was categorized into 4 grades according to Chirieac et al^18^: TRG 1, no residual carcinoma; TRG 2, 1% to 10% residual carcinoma; TRG 3, 11% to 50% residual carcinoma; TRG 4, >50% residual carcinoma.

### Follow-up

The protocol for postoperative follow-up was based on Dutch clinical guidelines and the SANO study protocol.^11,12^ Patients visited the outpatient clinic every 3 months in the first year, every 4 months in the second year, every 6 months in the third year, and yearly thereafter until the fifth year. Beyond 5 years, patients only visited the outpatient clinic in the case of symptoms. General practitioners of patients who had not visited the outpatient clinic beyond 2 years were contacted to provide a complete recording of relapse, most recent follow-up status, and cause of death, if applicable. During follow-up, diagnostic procedures were only performed when considered clinically necessary.^11,12^

### Outcomes

The primary outcome was overall survival, defined as the interval between the date of diagnosis and the date of all-cause death or last follow-up. Secondary outcomes were disease-free survival, resection rate, R0 resection rate, TRG, ypN stage, and postoperative complications. Disease-free survival was defined as the interval between the date of diagnosis and the date of disease progression, all-cause death, or last follow-up, whichever occurred first. The resection rate was defined as the proportion of patients in whom an esophagectomy was performed and excluded patients who underwent surgery without resection of the esophagus. Postoperative complications were defined according to Esophagectomy Complications Consensus Group definitions and graded according to the Clavien-Dindo classification.^19,20^ Major complications were defined as Clavien-Dindo grade ≥IIIb.

### Time to Surgery

Patients were retrospectively stratified based on TTS >12 weeks (TTS>12w) and TTS ≤12 weeks (TTS≤12w). TTS was defined as the interval between the date of completion of nCRT (ie, last fraction of radiotherapy) and the date of surgery. The cutoff of 12 weeks was based on the preSANO and SANO trial rationale. In these trials, surgery is postponed in patients with clinically complete response 6 weeks after completion of nCRT, and instead, those patients undergo a second clinical response evaluation 10 to 12 weeks after completion of nCRT. Prolonged time to surgery could be the result of various reasons like logistics, additional diagnostic testing, or patient unfitness for surgery. Delaying surgery up to 12 weeks has been proven to be safe and does not affect disease-free or overall survival.^5,21,22^ Yet it is still unknown whether delaying surgery up to 12 weeks, if necessary, is safe for patients with clinically proven residual disease. Hence, surgery beyond 12 weeks was considered delayed surgery in this study.

### Statistical Analysis

Continuous variables are presented as means with SD and compared with Student *t* test or as medians with interquartile ranges (IQR) and compared with Kruskal-Wallis test for non-normally distributed values. Categorical variables are presented as proportions with percentages and compared by using the χ^2^ test. Fisher exact test is used in case of expected cell counts of <5 or in case 2 categorical variables were compared. Median follow-up time is calculated using the reverse Kaplan-Meier method. Overall survival and disease-free survival are calculated using the Kaplan-Meier method and compared with the log-rank test and multivariable Cox proportional hazards models. Cox regression was preferred over propensity score matching to enable an analysis of all included patients.^23^ The other secondary outcomes are compared with multivariable binary logistic regression for outcomes with 2 categories and ordinal logistic regression for outcomes with more than 2 categories. All multivariable models are adjusted for age, cT, cN, Charlson comorbidity index, WHO performance score after completion of nCRT, and weight loss during nCRT. No formal power analysis and sample size calculation were performed due to the inability to enroll more patients within this study design.

Statistical significance was defined as *P*<0.05 (two sided). Missing data for the primary and secondary outcomes is quantified as percentages in the tables. All analyses were performed using the “tableone,” “stats,” “car,” “MASS,” and “survival” packages in R (R Core Team, R Foundation for Statistical Computing, Vienna, Austria, version 3.6.1).

## RESULTS

### Demographic and Clinical Characteristics

**FIGURE 1:**
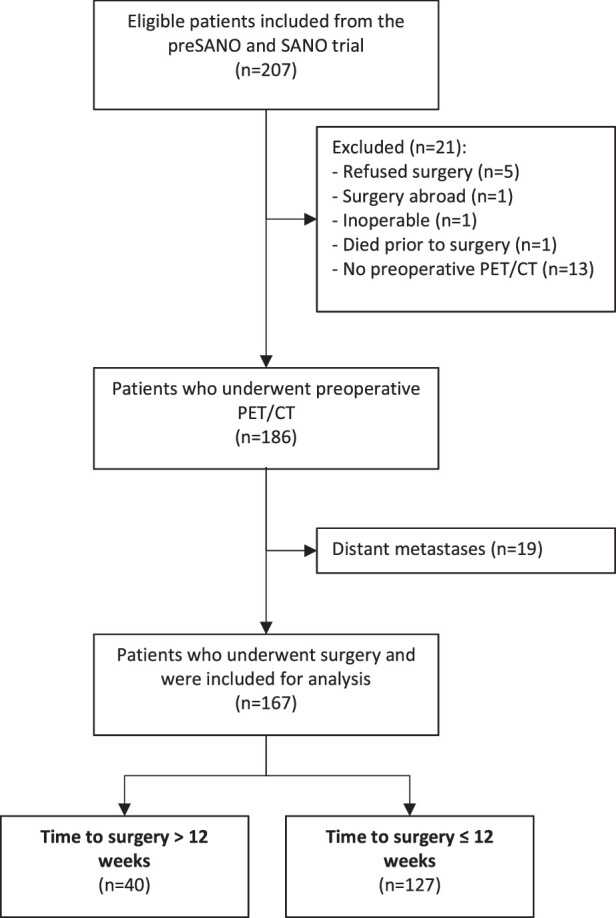
Flowchart of patients who had residual tumor in endoscopic biopsies take 4 to 6 weeks after completion of neoadjuvant chemoradiotherapy according to the CROSS regimen.

A total of 207 eligible patients were identified, of whom 167 met the inclusion criteria. A flowchart of the study is shown in Figure 1. Forty patients were included in the TTS>12w group and 127 in the TTS≤12w group. Of those in the TTS>12w group, 14 (35%) had a prolonged TTS due to logistic reasons (mostly waiting time), 13 (33%) had temporarily inoperable physical condition, 8 (20%) required additional diagnostic test(s), and 5 (13%) due to patient’s preference. Demographic and clinical characteristics are shown in Table 1. In short, weight loss during nCRT was higher in TTS>12w group compared with TTS≤12w. Other variables were well balanced. Post hoc subgroup analyses for adenocarcinoma histology are summarized in Supplementary Tables 3, 4, Supplemental Digital Content 1, http://links.lww.com/SLA/F263 and Supplementary Figures 1 and 2, Supplemental Digital Content 1, http://links.lww.com/SLA/F263 and show similar results in terms of primary outcomes.

**TABLE 1:** Demographic and Clinical Characteristics Stratified by Time to Surgery (TTS) >12 Weeks and ≤12 Weeks

	TTS >12 weeks	TTS ≤12 weeks	*P*
n	40	127	—
Age, median [IQR]	70 [60–73]	67 [61–72]	0.37
Sex, n (%)	—	—	1.00
Female	4 (10.0)	15 (11.8)	—
Male	36 (90.0)	112 (88.2)	—
BMI, median [IQR]	26.0 [23.7–29.1]	25.3 [23.4–27.9]	0.40
Tumor histology, n (%)	—	—	1.00
Adenocarcinoma	37 (92.5)	114 (89.8)	—
Squamous cell carcinoma	2 (5.0)	9 (7.1)	—
Other	1 (2.5)	4 (3.1)	—
Tumor length in cm, median [IQR]	5 [3-7]	5 [4-7]	0.53
cT stage, n (%)*	—	—	0.65
cT2	10 (25.0)	26 (20.5)	—
cT3	29 (72.5)	90 (70.9)	—
cT4	0 (0.0)	6 (4.7)	—
cTx	1 (2.5)	5 (3.9)	—
cN stage, n (%)*	—	—	0.50
cN0	17 (42.5)	40 (31.5)	—
cN1	14 (35.0)	53 (41.7)	—
cN2	8 (20.0)	30 (23.6)	—
cN3	0 (0.0)	3 (2.4)	—
cNx	1 (2.5)	1 (0.8)	—
Charlson Comorbidity Index, n (%)	—	—	0.59
CCI 0	1 (2.5)	3 (2.4)	—
CCI 1	6 (15.0)	17 (13.4)	—
CCI 2	6 (15.0)	31 (24.4)	—
CCI 3	9 (22.5)	38 (29.9)	—
CCI 4	9 (22.5)	19 (15.0)	—
CCI 5	5 (12.5)	9 (7.1)	—
CCI ≥6	4 (10.0)	10 (7.9)	—
WHO performance score after completion of nCRT, n (%)	—	—	0.35
WHO 0	16 (40.0)	45 (35.4)	—
WHO 1	18 (45.0)	72 (56.7)	—
WHO 2	5 (12.5)	8 (6.3)	—
WHO 3	1 (2.5)	2 (1.6)	—
Neoadjuvant chemoradiotherapy			
No. completed cycles nCT, median [IQR]	5 [5–5]	5 [5–5]	0.56
Total nRT dose (Gy), median [IQR]	41.4 [41.4-41.4]	41.4 [41.4-41.4]	0.66
Weight loss in kg, median [IQR]†	7 [4–11]	5 [2–8]	**0.020**
Time to biopsies in weeks, median [IQR]‡	6.1 [5.7–7.1]	5.4 [4.9–5.9]	**<0.001**
Time to PET/CT in weeks, median [IQR]‡	8.5 [7.6–10.2]	6.9 [6.4–7.7]	**<0.001**
Time to surgery in weeks, median [IQR]‡	13.7 [12.7–15.9]	9.7 [8.7–10.6]	**<0.001**
Time from PET/CT to surgery in weeks, median [IQR]	5.0 [4.0–6.0]	2.0 [1.0–3.0]	**<0.001**

* According to the 7th edition of the Union for International Cancer Control TNM Staging Manual.

† Measured as the difference in weight between 3 months before diagnosis and the last session of nCRT.

‡ Measured as the difference between the last day of radiotherapy and the day of the intervention (endoscopic biopsies, PET/CT or surgery).

The median time from completion of nCRT to preoperative PET/CT scan was 8.5 weeks in the TTS>12w group [IQR 7.6–10.2] and 6.9 weeks in the TTS≤12w group ([IQR 6.4–7.7], *P*<0.001). The median time biopsies was 6.1 weeks in the TTS>12w group [IQR 5.7–7.1] and 5.4 weeks in the TTS≤12w group ([IQR 4.9–5.9], *P*<0.001). Median time to surgery was 13.7 weeks in the TTS>12w group [IQR 12.7–15.9] and 9.7 [IQR 8.7–10.6] weeks in the TTS≤12w group (*P*<0.001). Median time from the preoperative PET/CT scan to surgery was 5.0 weeks in de TTS>12w group [IQR 4.0–6.0] and 2.0 [IQR 1.0–3.0] weeks in the TTS≤12w group (*P*<0.001).

### Survival

**FIGURE 2:**
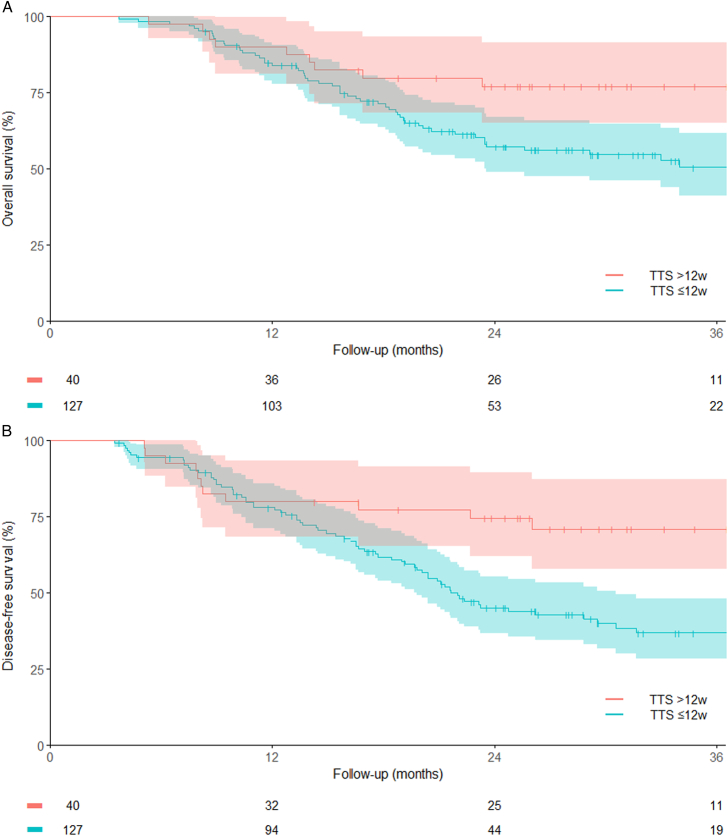
Overall survival (A) and disease-free survival (B) with 95% CIs of patients with histologically proven residual tumor 6 weeks after completion of nCRT, stratified by time to surgery (TTS) of >12 weeks and ≤12 weeks after completion of nCRT.

Median follow-up was 30.0 months [IQR 24.0–38.9], respectively 29.6 months [IQR 22.9–38.5], and 30.4 months [IQR 25.4–44.5] in the TTS>12w group and TTS≤12w group. Unadjusted overall and disease-free survival curves are shown in Figure 2. TTS>12w was associated with significantly better overall survival (3-y survival probability of 77.7% [95% CI: 65.1–91.5] vs 50.5% [95% CI: 41.1–61.9], adjusted hazard ratio 0.46, 95% CI: 0.24–0.90, *P*=0.023) and disease-free survival (3-y disease-free survival probability of 77.0% (95% CI: 64.9–91.5) vs 50.9% (95% CI: 41.9–61.9), adjusted hazard ratio 0.48, 95% CI: 0.24–0.94, *P*=0.031) compared with TTS≤12w. Complete multivariable Cox regression models are shown in Supplementary Table 1 and 2, Supplemental Digital Content 1, http://links.lww.com/SLA/F263. Post hoc subgroup analysis for prolonged TTS due to unfitness did not show an increase in complications in the TTS>12w group, shown in Supplementary Table 5, 6, Supplemental Digital Content 1, http://links.lww.com/SLA/F263 and Figure 3, Supplemental Digital Content 1, http://links.lww.com/SLA/F263. Post hoc survival curve of TTS>12w vs TTS<10w, respectively, TTS of median 13.6 weeks [IQR 12.5–15.8] versus TTS of median 8.9 weeks ([IQR 8.1–9.7], *P*<0.001), is shown in Supplementary Figure 4, Supplemental Digital Content 1, http://links.lww.com/SLA/F263. Post hoc survival curve of patients with metastatic disease on preoperative PET/CT is shown in Supplementary Figure 5, Supplemental Digital Content 1, http://links.lww.com/SLA/F263.

### Surgery and Pathology

Esophagectomy was not performed due to intraoperative detected T4b or metastatic disease in four of 40 patients (10.0%) in the TTS>12w group and 5 of 127 patients (3.9%) in the TTS≤12w group (Table 2). Metastases were detected intraoperatively in 3 and 4 patients in the TTS>12w and TTS≤12w group, respectively, while T4b occurred in one patient in both groups. There were no statistical differences in resection rate, after adjusting for confounders, between the TTS>12w and TTS≤12w group (aOR 0.29, 95% CI: 0.05–1.56, *P*=0.14) (Table 3). The majority of all patients underwent a minimally invasive transthoracic resection. Anastomotic location and duration of surgery were comparable between both groups. R0 resection rate, TRG stage, ypT, and ypN stage were also comparable between both groups (Tables 2 and 3).

**TABLE 2:** Surgical Characteristics, Pathologic Characteristics and Postoperative Complications of Patients Who Underwent Resection Stratified by Time to Surgery (TTS) >12 Weeks and ≤12 Weeks

	TTS >12 weeks	TTS ≤12 weeks	*P*
n	40	127	
Resection, n (%)*	36 (90.0)	122 (96.1)	0.22
Surgical approach, n (%)			0.23
Transthoracic	26 (72.2)	101 (82.8)	
Transhiatal	10 (27.8)	21 (17.2)	
Surgical technique, n (%)			0.46
Totally minimally invasive	26 (72.2)	87 (71.3)	
Hybrid minimally invasive	9 (25.0)	24 (19.7)	
Open	1 (2.8)	11 (9.0)	
Location of anastomosis, n (%)			1.00
Cervical	17 (47.2)	58 (47.5)	
Thoracic	19 (52.8)	64 (52.5)	
Duration of surgery in minutes, median [IQR]	330 [245–355]	310 [246–381]	0.80
R0 resection, n (%)†	33 (91.7)	111 (91.0)	1.00
Tumor regression grade, n (%)‡			0.98
TRG 1	1 (2.8)	5 (4.1)	
TRG 2	8 (22.2)	27 (22.1)	
TRG 3	12 (33.3)	42 (34.4)	
TRG 4	15 (41.7)	48 (39.3)	
ypT stage, n (%)§	—	—	0.80
ypT0	1 (2.8)	5 (4.1)	—
ypT1	8 (22.2)	23 (18.9)	—
ypT2	11 (30.6)	28 (23.0)	—
ypT3	16 (44.4)	65 (53.3)	—
ypT4	0	1 (0.8)	—
ypN stage, n (%)§	—	—	1.00
ypN0	19 (52.8)	65 (53.3)	—
ypN1	8 (22.2)	26 (21.3)	—
ypN2	7 (19.4)	23 (18.9)	—
ypN3	2 (5.6)	8 (6.6)	—
Overall complications, n (%)	32 (88.9)	88 (72.1)	**0.046**
Major complications, n (%)∥	8 (25.0)	13 (14.8)	0.28
Respiratory complication, n (%)	27 (75.0)	51 (41.8)	**0.001**
Pneumonia, n (%)	17 (47.2)	34 (27.9)	**0.042**
Pulmonary embolism, n (%)	0 (0.0)	2 (1.6)	1.00
Cardiac complications, n (%)	7 (19.4)	27 (22.1)	0.82
Anastomotic leakage, n (%)	9 (25.0)	25 (20.5)	0.65
Chyle leakage, n (%)	7 (19.4)	8 (6.6)	**0.045**
Vocal cord dysfunction, n (%)	1 (2.8)	5 (4.1)	1.00
Conduit necrosis, n (%)	0 (0.0)	1 (0.8)	1.00
In-hospital and/or 90-day mortality, n (%)	1 (2.8)	4 (3.3)	1.00

* Patients who did not undergo resection included patients who had intraoperative detection of distant metastases (M1) or who had invasion in surrounding structures (T4b).

† A microscopically radical resection (R0) was defined as a histologically confirmed tumor-free proximal, distal and circumferential resection margin (margin>1 mm not required).

‡ According to Chirieac et al^18^: TRG 1, no residual carcinoma; TRG 2, 1-10% residual carcinoma; TRG 3, 11%–50% residual carcinoma; TRG 4, >50% residual carcinoma.

§ According to the seventh edition of the Union for International Cancer Control TNM Staging Manual.

∥ A major complication was defined as Clavien-Dindo grade ≥IIIb.

**TABLE 3:** Multivariable Models Comparing the Resection Rate Postoperative Complications and Pathologic Characteristics of Patients With Time to Surgery (TTS) >12 Weeks and ≤12 Weeks

TTS >12 weeks vs TTS ≤12 weeks	Adjusted odds ratio (95% CI)*	*P*
Resection†	0.29 (0.05–1.56)	0.14
R0 resection‡	0.94 (0.24–2.98)	0.92
Tumor regression grade§	1.42 (0.68–2.97)∥	0.35
ypN stage¶	1.13 (0.52–2.46)∥	0.75
Overall complication	3.16 (0.92–10.8)	0.06
Major complication#	2.12 (0.69–6.33)	0.18
Respiratory complication	3.66 (1.52–9.59)	**0.005**
Pneumonia	1.78 (0.76–4.11)	0.18
Pulmonary embolism	NA**	1.00 ¥
Cardiac complications	0.64 (0.21–1.75)	0.40
Anastomotic leakage	1.55 (0.57–3.99)	0.37
Chyle leakage	3.75 (1.12–12.8)	**0.031**
Vocal cord dysfunction	0.29 (0.01–2.47)	0.32
Conduit necrosis	NA**	1.00 ¥
In-hospital and/or 90-d mortality	0.88 (0.08–9.45)	0.92

* Adjusted for age, cT, cN, Charlson Comorbidity Index, WHO performance score after completion of nCRT, and weight loss during nCRT.

† Patients who did not undergo resection included patients who had intraoperative detection of distant metastases (M1) or who had invasion in surrounding structures (T4b).

‡ A microscopically radical resection (R0) was defined as a histologically confirmed tumor-free proximal, distal, and circumferential resection margin (margin>1 mm not required).

§ According to Chirieac et al^18^: TRG 1, no residual carcinoma; TRG 2, 1% to 10% residual carcinoma; TRG 3, 11% to 50% residual carcinoma; TRG 4, >50% residual carcinoma.

∥ Proportional odds ratios across categories.

¶ According to the seventh edition of the Union for International Cancer Control TNM Staging Manual.

# A major complication was defined as Clavien-Dindo grade ≥IIIb.

** Number of events insufficient for estimating odds ratio. The *P* value was calculated with Fisher exact test.

### Surgical Morbidity

In the TTS>12w group, 32 of 36 patients (88.9%) had at least 1 postoperative complication and 8 of 36 (25.0%) a major complication. In the TTS≤12w group, 88 of 122 patients (72.1%) had one or more postoperative complications and 13 of 122 (14.8%) had a major complication (Table 2).

The TTS>12w group had an increased risk of postoperative respiratory complications (aOR 3.66, 95% CI: 1.52–9.59, *P*=0.005) and chyle leakage (aOR 3.75, 95% CI: 1.12–12.8, *P*=0.031) compared with the TTS≤12w group. The risks of postoperative overall complications, major complications, pneumonia, pulmonary embolism, cardiac complications, anastomotic leakage, vocal cord dysfunction, conduit necrosis, and the 90-day and/or in-hospital mortality rate were comparable between both groups (Tables 2 and 3).

## DISCUSSION

In the present study, TTS of >12 weeks was associated with better overall and disease-free survival in patients with biopsy-proven residual disease 6 weeks after completion of nCRT. The risk of postoperative respiratory complications and chyle leakage was increased compared with those with TTS ≤12 weeks. TTS >12 weeks was not significantly correlated with a lower resection or R0 resection rate, nor with differences in TRG score, ypT or ypN stage.

On the basis of these results, time to surgery after nCRT, irrespective of response, does not seem to negatively affect survival, and could potentially be safely prolonged up to 12 weeks when necesary.^3–5,21,22,24–27^ However, results of the recent DICE study, an international multicenter retrospective cohort study, showed worse overall survival in patients with a TTS >50 days after completion of nCRT.^6^ The patient population of this cohort is not comparable to the present study, mainly because the majority of patients in the DICE study had a radiation dose of >50 Gy. The indication for this higher dosage and reason for delay, could not be retrieved from the paper, potentially introducing selection bias when comparing outcomes of prolonged TTS in initial nonoperative patients with patients that undergo higher radiation dose with curative intend, like standard of practice is the United States. The present study focuses on patients with postponed esophagectomy for residual disease after curative intended nCRT with 41.4 Gy (ie, trimodality treatment), which is correlated with better survival than salvage surgery after definitive chemoradiotherapy (for nonoperative patients) with doses of >50 Gy.^28,29^ This is supported by supplementary data of the DICE study, showing that the difference in overall survival is not present if patients treated with radiotherapy >50 Gy are excluded. Furthermore, patients in the DICE study who underwent postponed surgery had higher ASA scores compared with patients undergoing immediate surgery, also suggesting selection bias. Compared with the results of the recently published randomized NeoRes II trial, the present study observed a better overall survival in the prolonged TTS group.^4,5^ In the NeoRes II trial, overall survival was comparable between surgery after 4 to 6 weeks versus surgery after 10 to 12 weeks. In post hoc sub analyses, however, worse overall survival was reported for patients with TRG4 residual disease in the group with (prolonged) TTS at 10 to 12 weeks after nCRT. This sub analysis comprised only 20 patients for each group and the study was not designed for robust conclusions on survival for these subgroups. Eighteen patients (15%) did not undergo allocated esophagectomy in the prolonged TTS group, mostly due to intraoperatively or preoperatively detected metastatic disease. These patients were included in the primary (intention-to-treat) analysis, which is in contrast to the present study, where patients with preoperatively detected (interval) metastases could not be included due to unavailable data on time to surgery (per-protocol).

In the present study, increased survival in the TTS>12w group may be explained by (better) patient selection for surgery. In the TTS>12w group, the interval between completion of nCRT and the preoperative PET/CT was longer. This may have increased the chance of occult metastases to become detectable on preoperative PET/CT in patients with prolonged TTS, whom are excluded from analysis if metastases are detected due to missing TTS. Therefore, the TTS>12w group may contain less patients with (occult) metastatic disease. The time from PET/CT to surgery was also longer in the TTS>12w group, which may have led to (the nonsignificantly) higher rate of intraoperatively detected metastases. Given that these patients did not undergo a major resection while having metastases, this could have improved their survival as they would qualify faster for palliative chemo.^30^

The increase in postoperative respiratory complications and chyle leakage, and the trend in more overall postoperative complications, may reflect the poorer condition of patients in the TTS>12w group. Of all patients in the TTS>12w group, surgery was postponed in one-third due to reduced physical condition after completion of nCRT.^31^ While this study adjusted for confounders, these parameters may not have fully reflected the subtle differences in patients’ frailty. Nevertheless, the increase in postoperative respiratory complications and chyle leakage did not seem to affect postoperative and/or 90-day mortality nor overall and disease-free survival. The anastomotic leakage and chyle leakage rate are above benchmarking level since this data originates from a prospective trial, in a subgroup of patients with poor response to nCRT and prolonged TTS, which could explain the rate difference compared with benchmarks.

In the DICE study, no outcomes on postoperative complications were published, but the 90-day mortality might reflect increased salvage surgery-related complications.^6,28,29^ The authors of the NeoRes II trial hypothesized that postoperative morbidity and mortality may be higher around the end of the nCRT regimen, and thus a longer recovery period after nCRT may decrease the risk of postoperative complications.^4^ However, no differences in any of the postoperative complications nor in 90-day mortality were observed between TTS of four to 6 weeks versus TTS of 10 to 12 weeks. In the present study, it was hypothesized that patients with (substantial) residual tumor and a longer interval between nCRT and surgery may have an increased risk of postoperative complications, since the longer interval may allow growth of the residual tumor or an increase in post radiation fibrosis.^32,33^ Nevertheless, resection rate, R0 resection rate, TRG stage, as well as ypN stage were comparable between the TTS>12w and TTS≤12w group. This indicates comparable tumor residues and contradicts this hypothesis.

Strengths of the present study are the strict protocol for the clinical response evaluation and the use of prospective clinical trial data. Limitations of this study include that the retrospective nature of the analyses allowed for selection bias. Also, immortal time bias has been introduced due to the nonrandomized assignment to groups with different time intervals from completion of nCRT to surgery. However, only 1 patient who was eligible for inclusion died while awaiting surgery, thus limits the effect of immortal time bias on the outcome of this study. Furthermore, the staging modalities used in the clinical response evaluations by different physicians in different centers, and the conversion from regular biopsies to bite-on-bite biopsy technique for the detection of residual disease could have increased the interoberserver variability. However, the introduction of the bite-on-bite biopsy technique, and the combination of staging modalities improved the detection of residual disease significantly.^13,15,34,35^ The small sample size in the TTS>12w group could have led to a reduced statistical power for adequate comparison, especially in the analysis of resection rate and overall postoperative complications. Despite the small sample size in the TTS>12w group, however, a significant advantage in overall and disease-free survival was found compared with TTS ≤12 weeks.

In conclusion, this study suggests that esophagectomy in patients with histologically proven residual disease after completion of nCRT can be postponed when necessary and does not seem to negatively affect the overall and disease-free survival. Postponed esophagectomy was, however, associated with an increased risk of postoperative complications. Future research should focus on the optimal timing of preoperative restaging and optimizing adequate recovery from nCRT, as well as on identifying predictive factors for postoperative complications and prehabilitation programs to prevent them.

## Supplementary Material
