# Supplementary material for: Prolonged Time to Surgery in Patients With Residual Disease After Neoadjuvant Chemoradiotherapy for Esophageal Cancer
Source: Ann Surg. 2024 Aug 13;283(2):268–76. doi: 10.1097/SLA.0000000000006488 (PMC12783346; doi:10.1097/SLA.0000000000006488)
Supplement: Supplementary file 1 [file sla-283-268-s001.docx]

**Supplementary content**

**Supplementary Table 1.** Multivariable Cox regression model for overall survival.

| **Factor** | **Adjusted hazard ratio (95% CI)*** | **p** |
| --- | --- | --- |
| Group |  |  |
| TTS ≤12 weeks | Reference |  |
| TTS >12 weeks | 0.46 (0.24-0.90) | **0.023** |
| Age | 1.01 (0.96-1.07) | 0.64 |
| cT stage** |  |  |
| cT2 | Reference |  |
| cT3 | 2.55 (1.11-5.84) | **0.027** |
| cT4 | 1.69 (0.32-9.01) | 0.54 |
| cTx | 3.97 (1.00-15.7) | **0.049** |
| cN stage** |  |  |
| cN0 | Reference |  |
| cN1 | 0.99 (0.53-1.84) | 0.97 |
| cN2 | 0.74 (0.35-1.58) | 0.44 |
| cN3 | 2.12 (0.46-9.79) | 0.34 |
| cNx | N/A‡ | 1.00‡ |
| Charlson Comorbidity Index |  |  |
| CCI 0 | Reference |  |
| CCI 1 | 0.80 (0.17-3.83) | 0.78 |
| CCI 2 | 0.36 (0.06-1.99) | 0.24 |
| CCI 3 | 0.41 (0.06-2.67) | 0.35 |
| CCI 4 | 0.68 (0.10-4.52) | 0.69 |
| CCI 5 | 0.40 (0.05-3.44) | 0.41 |
| CCI ≥6 | 0.23 (0.03-1.99) | 0.18 |
| WHO performance score after completion of nCRT |  |  |
| WHO 0 | Reference |  |
| WHO 1 | 2.01 (1.07-3.77) | **0.030** |
| WHO 2 | 2.83 (1.00-8.07) | 0.05 |
| WHO 3 | 2.94 (0.61-14.2) | 0.18 |
| Weight loss† | 1.05 (0.99-1.10) | 0.05 |
| * Adjusted for age, cT, cN, Charlson Comorbidity Index, WHO performance score after completion of nCRT, and weight loss during nCRT.  ** According to the 7^th^ ed. of the Union for International Cancer Control TNM Staging Manual.  † Measured as the difference in weight between 3 months prior to diagnosis and the last session of nCRT.  ‡ Number of events insufficient for estimating hazard ratio. The p-value was calculated with Fisher’s exact test. | | |

**Supplementary Table 2.** Multivariable Cox regression model for disease-free survival.

| **Factor** | **Adjusted hazard ratio (95% CI)*** | **p** |
| --- | --- | --- |
| Group |  |  |
| TTS ≤12 weeks | Reference |  |
| TTS >12 weeks | 0.48 (0.24-0.94) | **0.031** |
| Age | 1.01 (0.96-1.06) | 0.73 |
| cT stage** |  |  |
| cT2 | Reference |  |
| cT3 | 2.58 (1.13-5.87) | **0.024** |
| cT4 | 2.05 (0.40-10.6) | 0.39 |
| cTx | 3.06 (0.79-11.9) | 0.11 |
| cN stage** |  |  |
| cN0 | Reference |  |
| cN1 | 1.02 (0.55-1.91) | 0.95 |
| cN2 | 0.82 (0.39-1.74) | 0.61 |
| cN3 | 2.14 (0.46-9.90) | 0.33 |
| cNx | N/A‡ | 1.00‡ |
| Charlson Comorbidity Index |  |  |
| CCI 0 | Reference |  |
| CCI 1 | 0.96 (0.20-4.66) | 0.96 |
| CCI 2 | 0.45 (0.08-2.55) | 0.37 |
| CCI 3 | 0.47 (0.07-3.06) | 0.43 |
| CCI 4 | 0.79 (0.12-5.18) | 0.80 |
| CCI 5 | 0.49 (0.06-4.26) | 0.52 |
| CCI ≥6 | 0.38 (0.04-2.86) | 0.32 |
| WHO performance score after completion of nCRT |  |  |
| WHO 0 | Reference |  |
| WHO 1 | 1.97 (1.05-3.67) | **0.033** |
| WHO 2 | 2.63 (0.93-7.45) | 0.07 |
| WHO 3 | 2.84 (0.59-13.7) | 0.19 |
| Weight loss† | 1.05 (1.01-1.10) | **0.045** |
| * Adjusted for age, cT, cN, Charlson Comorbidity Index, WHO performance score after completion of nCRT, and weight loss during nCRT.  ** According to the 7^th^ ed. of the Union for International Cancer Control TNM Staging Manual.  † Measured as the difference in weight between 3 months prior to diagnosis and the last session of nCRT.  ‡ Number of events insufficient for estimating hazard ratio. The p-value was calculated with Fisher’s exact test. | | |

**Supplementary Table 3.** Demographic and clinical characteristics for adenocarcinoma histology, stratified by time to surgery (TTS) >12 weeks and ≤12 weeks.

|  | **TTS >12 weeks** | **TTS ≤12 weeks** | **p** |
| --- | --- | --- | --- |
| n | 37 | 114 |  |
| Age, median [IQR] | 70 [60-73] | 67 [61-72] | 0.42 |
| Sex, n (%) |  |  | 0.75 |
| Female | 4 (10.8) | 10 (8.8) |  |
| Male | 33 (89.2) | 104 (91.2) |  |
| BMI, median [IQR] | 25.6 [23.3-29.1] | 25.5 [23.6-28.1] | 0.87 |
| Tumor histology, n (%) |  |  |  |
| Adenocarcinoma | 37 (100) | 114 (100) |  |
| Tumor length in cm, median [IQR] | 4 [3-7] | 5 [4-7] | 0.49 |
| cT stage, n (%)* |  |  | 0.86 |
| cT2 | 9 (24.3) | 24 (21.1) |  |
| cT3 | 27 (73.0) | 83 (72.8) |  |
| cT4 | 0 (0.0) | 4 (3.5) |  |
| cTx | 1 (2.7) | 3 (2.6) |  |
| cN stage, n (%)* |  |  | 0.38 |
| cN0 | 17 (45.9) | 37 (32.5) |  |
| cN1 | 13 (35.1) | 46 (40.4) |  |
| cN2 | 6 (16.2) | 27 (23.7) |  |
| cN3 | 0 (0.0) | 3 (2.6) |  |
| cNx | 1 (2.7) | 1 (0.9) |  |
| Charlson Comorbidity Index, n (%) |  |  | 0.51 |
| CCI 0 | 1 (2.7) | 3 (2.6) |  |
| CCI 1 | 5 (13.5) | 15 (13.2) |  |
| CCI 2 | 6 (16.2) | 30 (26.3) |  |
| CCI 3 | 9 (24.3) | 35 (30.7) |  |
| CCI 4 | 7 (18.9) | 16 (14.0) |  |
| CCI 5 | 5 (13.5) | 6 (5.3) |  |
| CCI ≥6 | 4 (10.8) | 9 (7.9) |  |
| WHO performance score after completion of nCRT, n (%) |  |  | 0.28 |
| WHO 0 | 15 (40.5) | 38 (33.3) |  |
| WHO 1 | 17 (45.9) | 67 (58.8) |  |
| WHO 2 | 5 (13.5) | 7 (6.1) |  |
| WHO 3 | 0 (0.0) | 2 (1.8) |  |
| Weight loss in kg, median [IQR] ** | 7 [4-11] | 5 [2-8] | **0.019** |
| Time to biopsies in weeks, median [IQR] † | 6.1 [5.7-7.0] | 5.4 [4.9-5.9] | **<0.001** |
| Time to PET/CT in weeks, median [IQR] † | 8.6 [7.9-10.0] | 6.9 [6.4-7.7] | **<0.001** |
| Time to surgery in weeks, median [IQR] † | 13.7 [12.7-15.9] | 9.7 [8.7-10.6] | **<0.001** |
| Time from PET/CT to surgery in weeks, median [IQR] | 5.0 [4.0-6.0] | 2.0 [1.0-3.0] | **<0.001** |
| * According to the 7^th^ ed. of the Union for International Cancer Control TNM Staging Manual.  ** Measured as the difference in weight between 3 months prior to diagnosis and the last session of nCRT.  † Measured as the difference between the last day of radiotherapy and the day of the intervention (endoscopic biopsies, PET/CT or surgery). | | | |

**Supplementary Table 4.** Surgical, pathological and complication characteristics for adenocarcinoma histology, stratified by time to surgery (TTS) >12 weeks and ≤12 weeks.

|  | **TTS >12 weeks** | **TTS ≤12 weeks** | **p** |
| --- | --- | --- | --- |
| n | 37 | 114 |  |
| Resection, n (%)* | 34 (91.9) | 109 (95.6) | 0.41 |
| Surgical approach, n (%) |  |  | 0.15 |
| Transthoracic | 24 (70.6) | 90 (82.6) |  |
| Transhiatal | 10 (29..4) | 19 (17.4) |  |
| Location of anastomosis, n (%)  Cervical  Thoracic | 16 (47.1)  18 (52.9) | 51 (46.8)  58 (53.2) | 1.00 |
| Surgical technique, n (%) |  |  | 0.41 |
| Totally minimally invasive  Hybrid minimally invasive  Open | 24 (70.6) 9 (26.5)  1 ( 2.9) | 78 (71.6)  21 (19.3)  10 ( 9.2) |  |
| R0 resection, n (%) ** | 31 (93.9) | 94 (86.2) | 0.89 |
| Tumor regression grade, n (%) † |  |  | 0.98 |
| TRG 1 | 1 (2.9) | 4 (3.7) |  |
| TRG 2 | 7 (20.6) | 23 (21.1) |  |
| TRG 3 | 11 (32.4) | 38 (34.9) |  |
| TRG 4 | 15 (44.1) | 44 (40.4) |  |
| ypT stage, n (%) ‡ |  |  | 0.90 |
| ypT0 | 1 (2.9) | 4 (3.7) |  |
| ypT1 | 8 (23.5) | 22 (20.2) |  |
| ypT2 | 10 (29.4) | 26 (23.9) |  |
| ypT3 | 15 (44.1) | 56 (51.4) |  |
| ypT4 | 0 (0.0) | 1 (0.9) |  |
| ypN stage, n (%) ‡ |  |  | 0.97 |
| ypN0 | 18 (52.9) | 55 (50.5) |  |
| ypN1 | 8 (23.5) | 24 (22.0) |  |
| ypN2 | 6 (17.6) | 22 (20.2) |  |
| ypN3 | 2 (5.9) | 8 (7.3) |  |
| Overall complications, n (%) | 31 (91.2) | 78 (71.6) | **0.021** |
| Major complications, n (%)¶ | 8 (25.8) | 12 (15.4) | 0.27 |
| Respiratory complication, n (%) | 26 (76.5) | 42 (38.5) | **0.001** |
| Pneumonia, n (%) | 16 (47.1) | 27 (24.8) | **0.018** |
| Pulmonary embolism, n (%) | 0 (0.0) | 1 (0.9) | 1.00 |
| Cardiac complications, n (%) | 7 (20.6) | 24 (22.0) | 1.00 |
| Anastomotic leakage, n (%) | 9 (26.5) | 24 (22.0) | 0.64 |
| Chyle leakage, n (%) | 7 (20.6) | 7 (6.4) | **0.023** |
| Vocal cord dysfunction, n (%) | 1 (2.9) | 5 (4.6) | 1.00 |
| Conduit necrosis, n (%) | 0 (0.0) | 1 (0.9) | 1.00 |
| In-hospital and/or 90-day mortality, n (%) | 1 (2.9) | 3 (2.8) | 1.00 |
| * Patients who did not undergo resection included patients who had intraoperative detection of distant metastases (M1) or who had invasion in surrounding structures (T4b).  ** A microscopically radical resection (R0) was defined as a histologically confirmed tumor-free proximal, distal and circumferential resection margin (margin>1mm not required).  † According to Chirieac et al.: TRG 1, no residual carcinoma; TRG 2, 1-10% residual carcinoma; TRG 3, 11-50% residual carcinoma; TRG 4, more than 50% residual carcinoma.  ‡ According to the 7^th^ ed. of the Union for International Cancer Control TNM Staging Manual.  ¶ A major complication was defined as Clavien-Dindo grade ≥IIIb. | | | |

**Supplementary Table 5.** Complications characteristics for TTS>12w subgroup, stratified by delay due to unfitness.

| Reason TTS>12 weeks | **Unfitness** | **Else** | **p** |
| --- | --- | --- | --- |
| n | **13** | **27** |  |
| Resection, n (%) * | 11 (84.6) | 25 (92.6) | 0.58 |
| Overall complications, n (%)  Major complications, n (%) ¶ | 11 (100)  3 (27.3) | 21 (84)  5 (23.8) | 0.29  1.00 |
| Respiratory complication, n (%)  Pneumonia, n (%)  Pulmonary embolism, n (%) | 9 (81.8)  7 (63.6)  0 (0.0) | 18 (72.0)  10 (40.0)  0 (0.0) | 0.69  0.28  1.00 |
| Cardiac complications, n (%) | 3 (27.3) | 4 (16.0) | 0.65 |
| Anastomotic leakage, n (%) | 2 (18.2) | 7 (28.0) | 0.69 |
| Chyle leakage, n (%) | 2 (18.2) | 5 (20.0) | 1.00 |
| Vocal cord dysfunction, n (%) | 0 (0.0) | 1 (4.0) | 1.00 |
| Conduit necrosis, n (%) | 0 (0.0) | 0 (0.0) | 1.00 |
| In-hospital and/or 90-day mortality, n (%) | 0 (0.0) | 1 (4.0) | 1.00 |
| * Patients who did not undergo resection included patients who had intraoperative detection of distant metastases (M1) or who had invasion in surrounding structures (T4b).  ¶ A major complication was defined as Clavien-Dindo grade ≥IIIb. | | | |

**Supplementary Table 6.** Complication characteristics, delay due to unfitness excluded, stratified by time to surgery (TTS) >12 weeks and ≤12 weeks.

| TTS>12 weeks due to unfitness excluded | **TTS<12w** | **TTS>12w** | **p** |
| --- | --- | --- | --- |
| n | **127** | **27** |  |
| Resection, n (%) * | 122 (96.1) | 25 (92.6) | 0.36 |
| Overall complications, n (%)  Major complications, n (%) ¶ | 88 (72.1)  13 (14.8) | 21 (84.0)  5 (23.8) | 0.32  0.33 |
| Respiratory complication, n (%)  Pneumonia, n (%)  Pulmonary embolism, n (%) | 51 (41.8)  34 (27.9)  2 (1.6) | 18 (72.0)  10 (40.0)  0 (0.0) | **0.008**  0.24  1.00 |
| Cardiac complications, n (%) | 27 (22.1) | 4 (16.0) | 0.60 |
| Anastomotic leakage, n (%) | 25 (20.5) | 7 (28.0) | 0.43 |
| Chyle leakage, n (%) | 8 (6.6) | 5 (20.0) | **0.047** |
| Vocal cord dysfunction, n (%) | 5 (4.1) | 1 (4.0) | 1.00 |
| Conduit necrosis, n (%) | 1 (0.8) | 0 (0.0) | 1.00 |
| In-hospital and/or 90-day mortality, n (%) | 4 (3.3) | 1 (4.0) | 1.00 |
| * Patients who did not undergo resection included patients who had intraoperative detection of distant metastases (M1) or who had invasion in surrounding structures (T4b).  ¶ A major complication was defined as Clavien-Dindo grade ≥IIIb. | | | |

**Supplementary Figure 1.** Kaplan-Meier curve of Overall survival adenocarcinoma subgroup


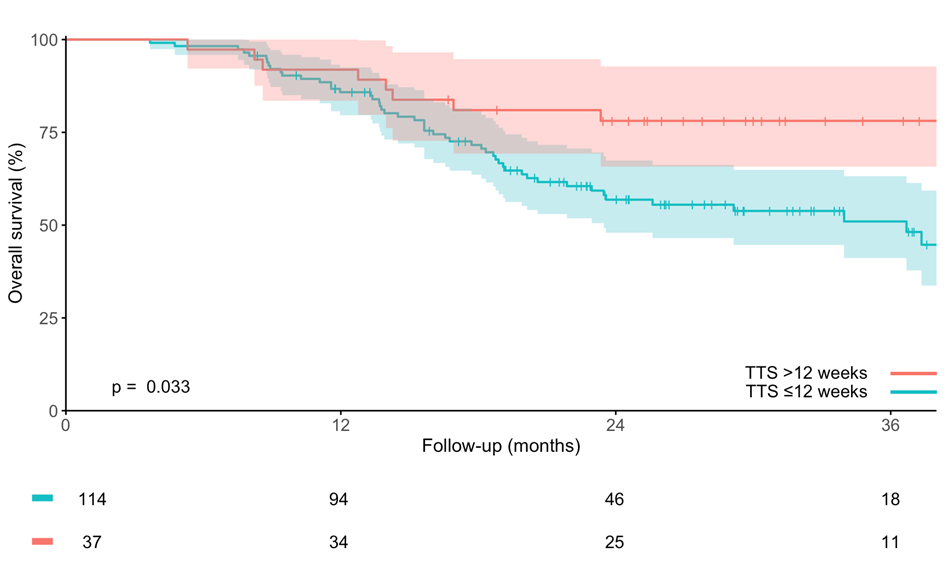

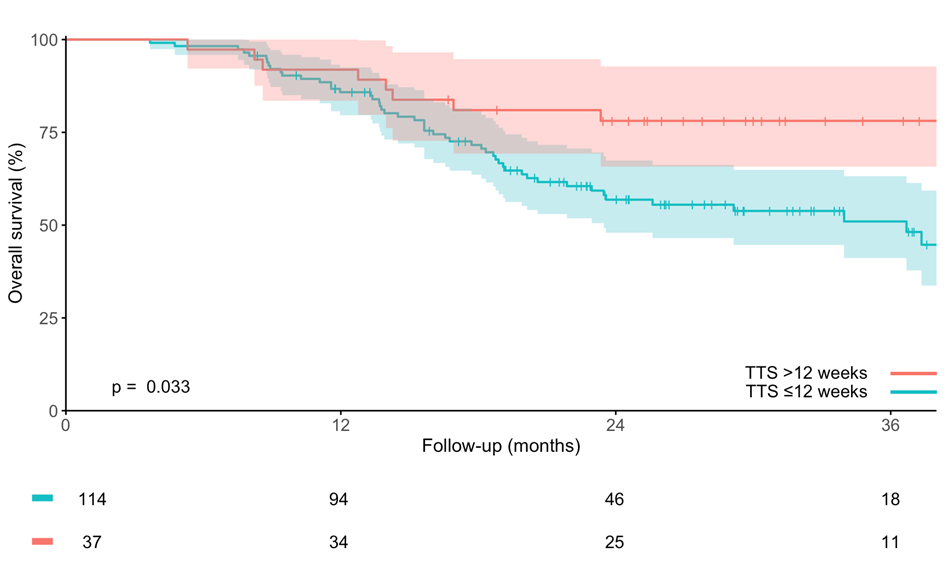


**Supplementary Figure 2.** Kaplan-Meier curve of Progression-free survival for adenocarcinoma subgroup


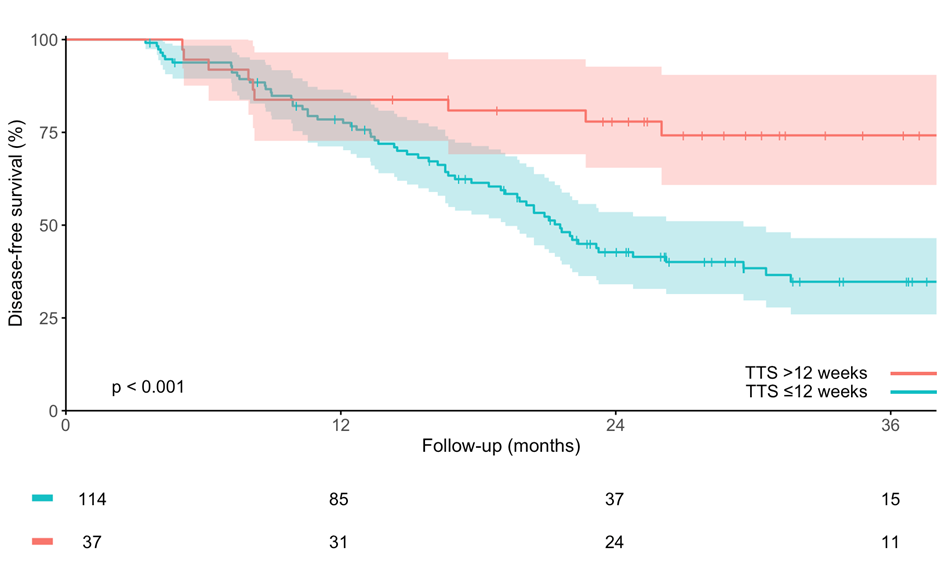

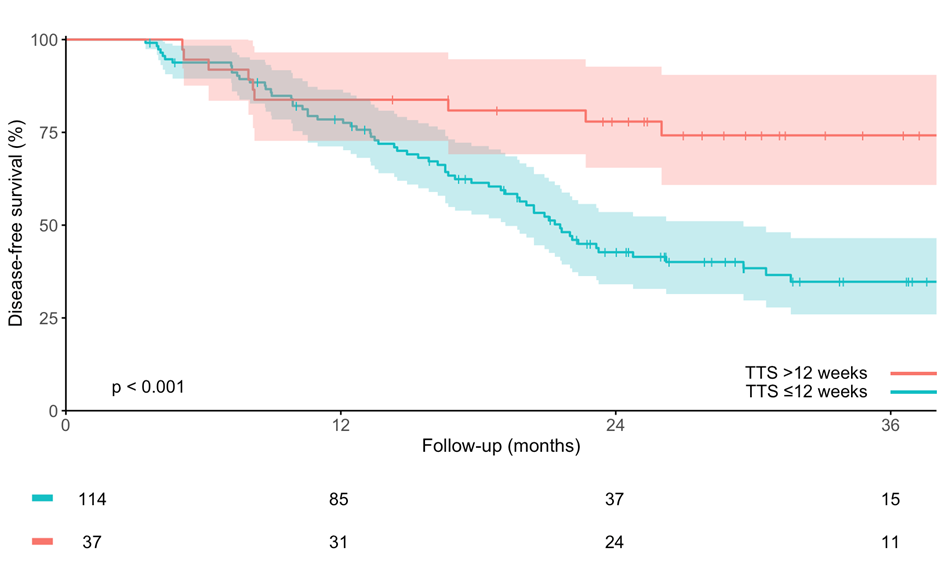


**Supplementary Figure 3.** Kaplan-Meier curve of TTS>12w subgroup, delay due to unfitness


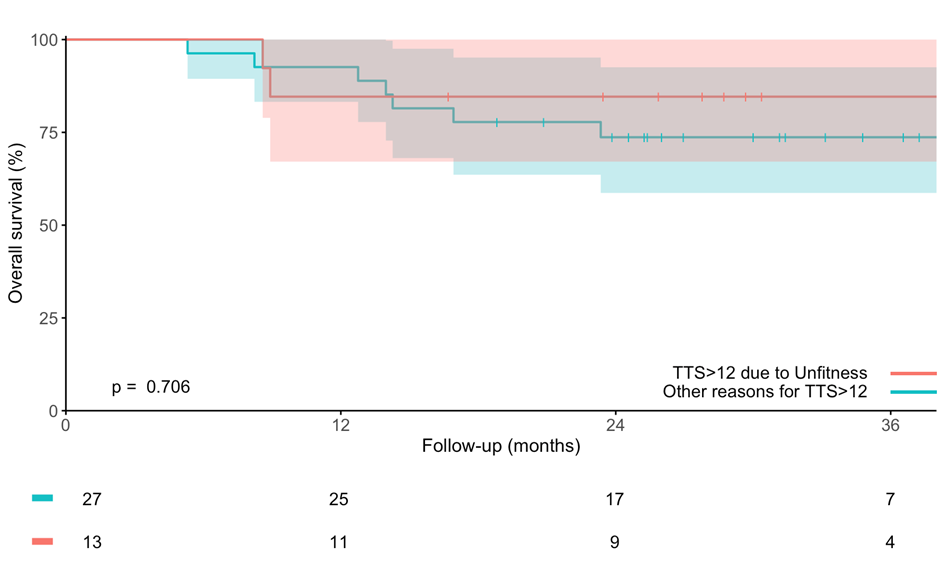

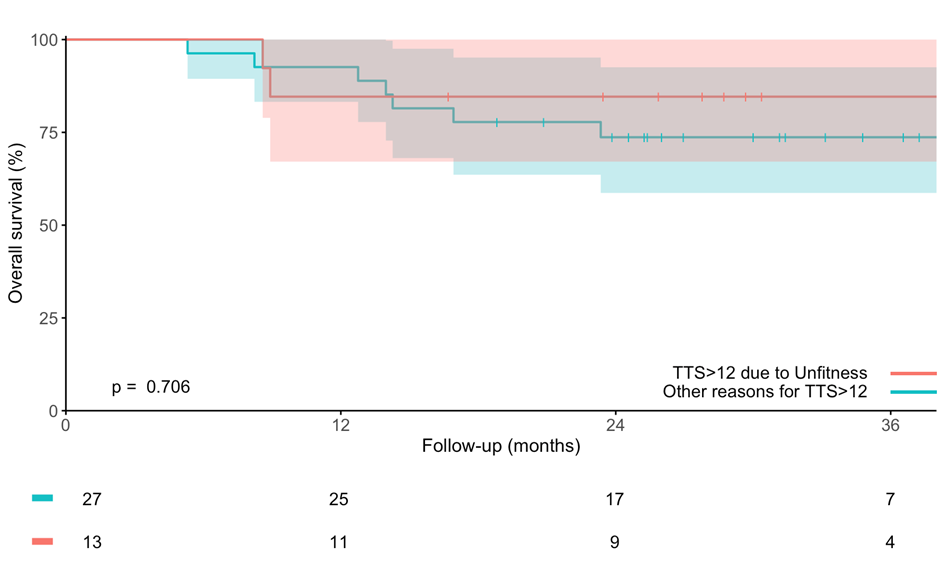


**Supplementary Figure 4.** Kaplan-Meier curve of TTS>12w versus TTS<10w subgroup.


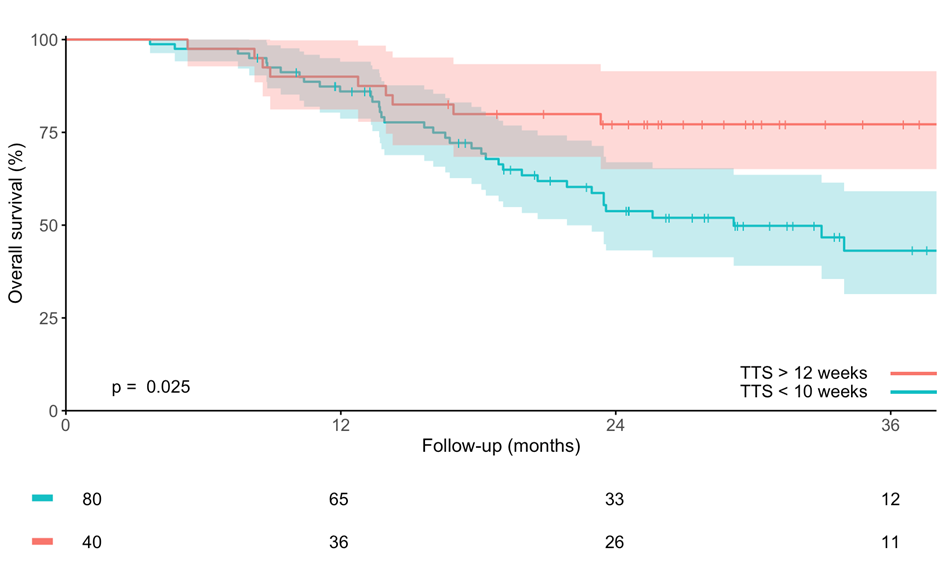

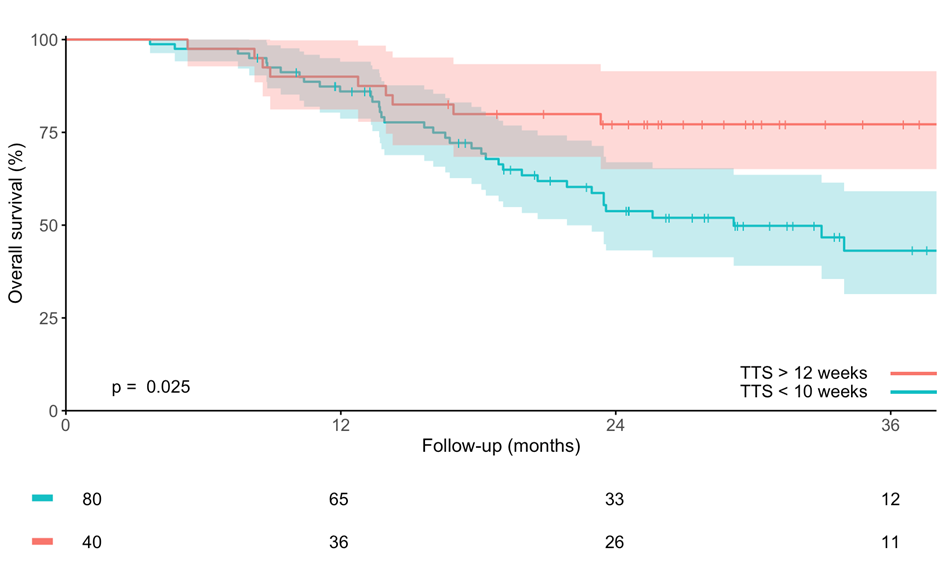


**Supplementary Figure 5.** Kaplan-Meier curve of patients with metastatic disease on preoperative PET/CT.

**
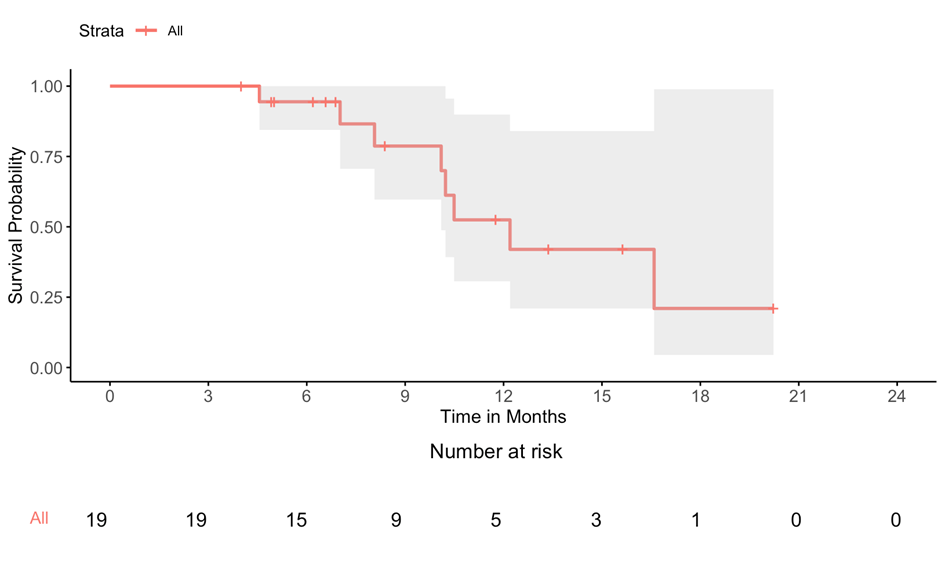
**
